# Gestión del proceso posanalítico en los laboratorios clínicos según los requisitos de la norma ISO 15189:2012. Consideraciones sobre la gestión de muestras clínicas, aseguramiento de la calidad en el proceso posanalítico y gestión de la información del laboratorio

**DOI:** 10.1515/almed-2020-0088

**Published:** 2021-05-21

**Authors:** Mª Liboria López Yeste, Antonia R. Pons Mas, Leonor Guiñón Muñoz, Silvia Izquierdo Álvarez, Fernando Marqués García, Aurora Blanco Font, Natalia F. Pascual Gómez, Lorena Sánchez Gancedo, Ana García Álvarez, Francisco A. Bernabeu Andreu, Mª Patrocinio Chueca Rodríguez, Luisa Álvarez Domínguez

**Affiliations:** Coordinadora de Calidad, CATLAB, Barcelona, España; Servicio de Análisis Clínicos, Hospital Universitari Son Espases, Mallorca, España; Directora de Calidad de los Laboratorios, Hospital de la Santa Creu i Sant Pau, Barcelona, España; Servicio de Bioquímica Clínica, Hospital Universitario Miguel Servet, Zaragoza, España; Servicio de Análisis Clínicos y Bioquímica Clínica, Laboratorio Clínico de la Metropolitana Norte, Hospital Universitario Germans Trias i Pujol, Badalona, Barcelona,España; Laboratori Clínic Territorial Metropolitana Sud, Hospital Universitari de Bellvitge, Barcelona, España; Servicio de Análisis Clínicos, Hospital Universitario de la Princesa, Madrid, España; Calidad, Instituto de Medicina Oncológica y Molecular, Asturias, España; Servicio Análisis Clínicos, Hospital Clínico San Carlos, Madrid, España; Servicio de Análisis Clínicos- Bioquímica Clínica, Hospital Universitario Puerta de Hierro, Madrid, España; Comisión de Acreditación de Laboratorios de la Sociedad Española de Medicina de Laboratorio, Tudela, Navarra, España; Comisión de Acreditación de Laboratorios de la Sociedad Española de Medicina de Laboratorio (SEQC^ML^), Barcelona, España

**Keywords:** acreditación, laboratorio clínico, norma ISO 15189, posanalítica, sistema de información del laboratorio

## Abstract

La norma ISO 15189:2012 exige una serie de requisitos en cuanto a la gestión de muestras clínicas, el aseguramiento de la calidad del proceso y la gestión de la información del laboratorio. Además, la entidad acreditadora, ENAC en España, tiene establecidas las condiciones para el uso de la marca en el informe de resultados del laboratorio acreditado. Las recomendaciones planteadas aplican a las actividades incluidas en el proceso posanalítico del laboratorio, así como al personal implicado. Se indican los criterios para que el laboratorio defina y documente el tiempo y las condiciones de retención de las muestras, para diseñar un control de calidad interno que verifique si las actividades posanalíticas alcanzan la calidad esperada, los requisitos que debe cumplir la gestión de la información y la necesidad de disponer de un plan de contingencia que asegure la comunicación de los resultados en todo momento. Asimismo, se describe el uso correcto de la marca de acreditación en los informes. Diversos gobiernos y sociedades científicas abogan por la obligatoriedad de la acreditación de los laboratorios clínicos. Siendo la norma ISO 15189 la más específica para demostrar su competencia técnica, es indispensable el conocimiento y la comprensión de sus requisitos para su correcta implantación.

## Introducción

Los laboratorios clínicos dedican cada vez más esfuerzo a mejorar sus habilidades metodológicas y de comunicación, con la finalidad de ayudar a interpretar los resultados analíticos. Además de los aspectos ya discutidos en un documento anterior [1] relacionados con la revisión, notificación y comunicación de los resultados, la norma UNE-EN ISO 15189:2013 (en adelante, norma ISO 15189) incluye otros requisitos a aplicar en el proceso posanalítico, que hacen referencia al almacenamiento, retención y desecho de las muestras, la inclusión del proceso posanalítico en el aseguramiento de la calidad y en la mejora continua del laboratorio, la gestión de la información, así como la necesidad de un plan de contingencia que asegure la comunicación de resultados en cualquier circunstancia [2], [3]. La ISO 15189 es una norma que se encuentra en el marco de las normas de buenas prácticas del laboratorio, utilizando la información que se obtiene en el seguimiento del sistema de gestión de la calidad para establecer las acciones de mejora adecuadas en cada momento.

La norma ISO 15189 provee un conjunto de requisitos cuyo cumplimento por parte del laboratorio induce a que éste aplique una metodología adecuada para la detección y la clasificación de los errores que se producen en el proceso posanalítico y adopte sistemas de trabajo y herramientas informáticas apropiadas para su reducción [4]. La edición vigente concede especial relevancia a la comunicación de los resultados, la gestión de la información del laboratorio y la gestión del riesgo. Asimismo, remarca el requisito de que el laboratorio disponga de planes de contingencia. Por otra parte, y teniendo en cuenta que el uso de la marca de ENAC en los informes es el medio por el cual las organizaciones acreditadas declaran públicamente el cumplimiento de todos los requisitos de acreditación, consideramos importante incluir un apartado sobre el documento de ENAC, CEA-ENAC-01 “Criterios para la utilización de la marca ENAC y la referencia a la condición de acreditado”, que establece las condiciones para su uso y que los laboratorios deben conocer (consultar la versión en vigor, disponible en la web de ENAC www.enac.es).

Este documento en ningún caso sustituye ni amplía la norma y ha de utilizarse como apoyo para su interpretación e implantación. Su ámbito de aplicación es el personal implicado y las actividades comprendidas en el proceso posanalítico del laboratorio clínico.

## Almacenamiento, retención y desecho de las muestras

**Figura 1:**
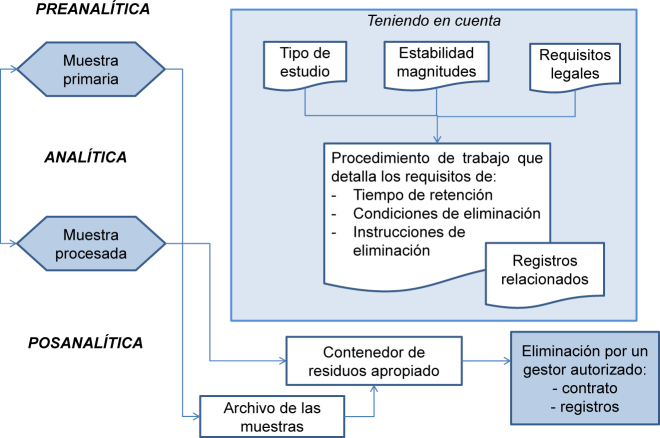
Esquema del proceso de almacenamiento, retención y desecho de muestras.

La norma ISO 15189 exige que el laboratorio defina y documente el tiempo de retención de las muestras, cómo se almacenan y eliminan. El procedimiento debe recoger el tiempo de retención y las condiciones de almacenamiento de las muestras, que se definirán en función de su naturaleza, de la estabilidad de las distintas magnitudes [5] y de cualquier requisito legal o reglamentario aplicable (ver Figura 1). Se debe considerar que los tiempos de conservación de las muestras, y sus registros, pueden ser mayores, atendiendo a aspectos de responsabilidad legal de ciertos tipos de estudios (por ejemplo, exámenes histológicos, genéticos o pediátricos).

Durante el almacenamiento, el acceso a las muestras debe estar restringido, a la vez que se asegura su trazabilidad y fácil localización para su reutilización si fuera necesario (para análisis adicionales, comprobación de resultados, requerimiento legal, etc.). Por ello, es recomendable registrar cualquier retirada de las muestras, la fecha y quién la retira, lo que es imprescindible en caso de requerimiento legal.

El laboratorio debe tener documentado el procedimiento de desecho de las muestras clínicas y de los consumibles, siendo responsabilidad del laboratorio el cumplimiento de la reglamentación y las recomendaciones vigentes en términos de prevención de riesgos laborales, aunque la retirada de residuos la lleve a cabo una empresa externa autorizada por él.

## Aseguramiento de la calidad y mejora continua

El proceso posanalítico, del mismo modo que los procesos preanalítico y analítico, debe evaluarse y someterse a auditorías para asegurar su conformidad y demostrar que se realiza de acuerdo a las necesidades y requisitos de los usuarios, además de mejorar su eficacia de forma continua.

Se deben tener en cuenta las actividades posanalíticas efectuadas, tanto intralaboratorio (fase posanalítica) como extralaboratorio (fase pos-posanalítica). Diferentes estudios han demostrado que en estas últimas suceden habitualmente el mayor número de errores, debido a la variedad de organizaciones y el elevado número de personas involucradas que no dependen directamente del laboratorio [6].

Se ha descrito que la incidencia de errores en el proceso posanalítico varía ampliamente, entre un 18% [7] y un 47% [8] de los errores totales. Los más frecuentes son la interpretación incorrecta de los resultados, la demora en la entrega de los informes, la pérdida del informe y la falta de notificación de incidencias con las muestras al médico responsable del paciente. Los errores posanalíticos pueden conllevar que el facultativo clínico tome una serie de decisiones equivocadas que se derivan de una mala interpretación de los resultados incluidos en el informe, decisiones que pueden afectar al curso clínico, al pronóstico y, como resultado final, a la salud del paciente. Se denominan habitualmente como errores en la fase o proceso pos-posanalítico [7], [8], [9]. La mejora de estos procesos es posible mediante la interacción con el personal de las distintas áreas asistenciales, donde la comunicación y la formación juegan un papel muy importante [10]. Otro problema que puede actuar como fuente de errores y que posiblemente se incrementará en el futuro, es la imposibilidad de consultar los resultados en la historia clínica electrónica por problemas informáticos.

**Figura 2:**
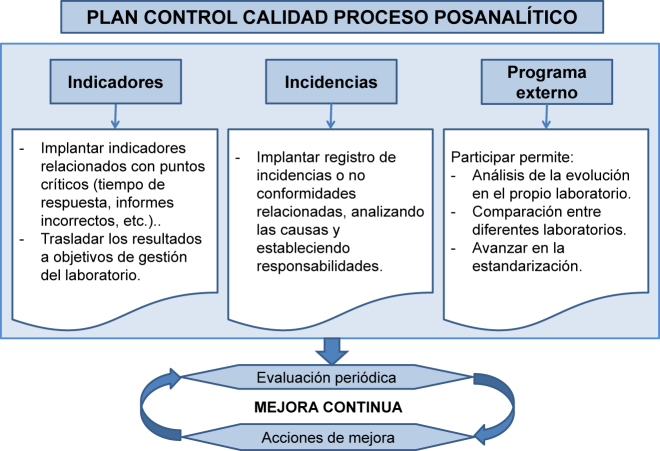
Características del plan de control de la calidad del proceso posanalítico.

El laboratorio debe diseñar un plan de control de calidad que verifique si las actividades posanalíticas alcanzan la calidad esperada. Este control se basará fundamentalmente en la definición de estrategias para la detección de errores y en el establecimiento de indicadores de la calidad (ver Figura 2).

Los indicadores de calidad de los procesos posanalíticos permiten evaluar de forma objetiva el servicio prestado y asegurar la calidad de este proceso en base a los objetivos de calidad establecidos. Para realizar una adecuada valoración de los resultados de los mismos, es fundamental establecer una comunicación regular, fluida y sistemática entre el personal del laboratorio y los facultativos asistenciales. Para la definición de indicadores del proceso posanalítico pueden considerarse aspectos como los intervalos de referencia, los puntos de corte y acción, la visualización gráfica, la autovalidación, los comentarios interpretativos, las pruebas reflejas (aquellas añadidas automáticamente en base a algoritmos) o reflexivas (añadidas por un profesional considerando el contexto clínico) [11], [12], la información clínica necesaria para poder interpretar los resultados correctamente, la comunicación de valores críticos y la transmisión efectiva de resultados de pruebas de laboratorio por el SIL [13]*.* De todos estos aspectos, el tiempo transcurrido entre la solicitud y la entrega del resultado, para muchos usuarios, es el principal indicador del desempeño de un laboratorio de análisis clínicos, lo que es especialmente relevante para los pacientes ingresados o atendidos en urgencias.

En la Conferencia de Consenso celebrada en Padua, en el año 2013, se emitió un modelo que se empezó a utilizar en el 2014 y que asigna una puntuación de prioridad a cada indicador, lo que ayuda a los laboratorios a introducir gradualmente mejoras de calidad en la práctica diaria. Por otro lado, se propuso un criterio para establecer las especificaciones de calidad para evaluar el rendimiento del laboratorio [13]. En la Conferencia de Padua de 2016 se revisaron los resultados obtenidos desde 2014 con el propósito de alcanzar un consenso sobre qué indicadores utilizar y qué especificaciones debían establecerse, con la finalidad de dar cumplimiento a requisitos de la norma, monitorizando la actividad crítica y minimizando el riesgo. Los indicadores establecidos evalúan todas las etapas posanalíticas, desde la validación de los resultados hasta su comunicación efectiva en el tiempo adecuado. Se expresan como el porcentaje de errores respecto a la actividad del laboratorio, siendo muy importante la forma de expresar dicha actividad (número de peticiones, de pacientes, de muestras, etc.), ya que ello permitirá́ la comparación con otros laboratorios o con las recomendaciones de las sociedades y organismos científicos. Los indicadores incluidos en el documento de consenso de Padua se detallan en la Tabla 1.

**Tabla 1:** Indicadores incluidos en el documento consenso de Padua del proceso posanalítico [15].

Actividad	Descripción	Fórmula de cálculo
Tiempos de respuesta	Número de informes emitidos fuera del tiempo pactado con respecto al número total de informes emitidos.	N^o^ informes fuera de tiempo en un año/n^o^ total de informes en un año × 100 (%).
Tiempos de respuesta	Tiempo de respuesta de distintas pruebas (potasio, INR, WBC, Troponina I ó T) desde su recepción en el laboratorio hasta la emisión del informe.	Día y hora de emisión – Día y hora de registro de entrada (días, horas, min) “… para el análisis no urgente de la concentración de potasio en suero”.
Informes de laboratorio corregidos	Porcentaje de informes corregidos por el laboratorio después de su emisión con respecto al número total de informes.	N^o^ informes corregidos en un año/n^o^ total de informes en un año × 100 (%).
Notificación de resultados críticos	Resultados críticos notificados fuera del tiempo establecido con respecto al total de resultados críticos notificados.	N^o^ resultados críticos fuera de tiempo en un año/ n^o^ total de resultados críticos en un año x 100 (%) “… para todos los análisis con indicación de notificación urgente; … para el análisis no urgente de la concentración de potasio en suero, etc.”.
Notificación de resultados críticos	Valor medio del tiempo que se tarda en comunicar los resultados críticos.	Promedio (hora de emisión de resultado – hora de comunicación de resultado a responsable del paciente (min)” “… para todos los análisis con indicación de notificación urgente; … para el análisis no urgente de la concentración de potasio en suero, etc.”.

Para más información puede consultarse el documento: https://www.ifcc.org/media/455725/Quality_Indicators_Key_Processes.pdf [15].

Para superar estos problemas e identificar el estado del arte sobre los errores que ocurren en el proceso total de laboratorio, el Grupo de Trabajo “Errores de Laboratorio y Seguridad del Paciente” (WG-LEPS) de la Federación Internacional de Química Clínica y Medicina de Laboratorio (IFCC), implementó en el año 2008 un proyecto destinado a definir un modelo común de indicadores (MQI), un método armonizado para la recopilación de datos, administrado como un Programa de Garantía de Calidad Externa (EQAP) en el que se garantiza la confidencialidad [14], [15].

El laboratorio deberá participar, asimismo, en algún programa de comparación interlaboratorios. La importancia de la participación en programas de evaluación externa estriba en que, a pesar de la falta de consenso actual sobre sus especificaciones de calidad, nos aportan información sobre si los resultados alcanzan las especificaciones fijadas en cada laboratorio y nos permiten compararnos con el resto de los participantes. A pesar del elevado número de errores que ocurren a lo largo del proceso posanalítico, y que la participación sea un requisito de la norma ISO 15189 (5.6.3), estos programas no están aún muy desarrollados. En los últimos tiempos diversos organismos los están poniendo en práctica en paralelo con los programas de intercomparación de las fases preanalítica y analítica. Entre estos se encuentra el programa de intercomparación diseñado por el Grupo de Trabajo WG-LEPS de la IFCC, activo desde el 2008, en el que se evalúa el modelo de indicadores diseñado por el mismo grupo. Aunque no es lo habitual, algunos programas de intercomparación y los que ofrecen RCPA QAP y UK NEQAS, abordan los comentarios interpretativos en el área de la Bioquímica Clínica [6].

## Gestión de la información del laboratorio

**Figura 3:**
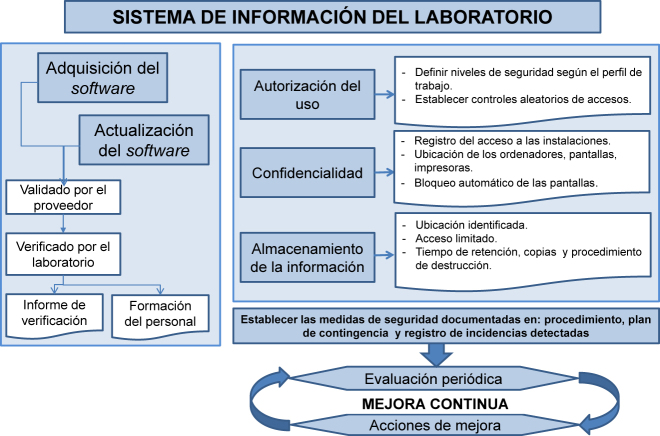
Principales requisitos de la gestión de la información del laboratorio.

Las nuevas tecnologías y el aumento del número de datos que gestiona el laboratorio han generado un incremento de los requisitos de los sistemas de gestión de la información de los laboratorios [16]. A este respecto, la norma indica que el laboratorio ha de disponer de un sistema de gestión de la información que proporcione accesibilidad, integridad, seguridad y confidencialidad a la información del paciente. Es importante que se tengan en cuenta tanto los equipos informáticos como los sistemas no informatizados, así como todas las situaciones en que la información es recibida, generada o transmitida. La Figura 3 resume los requisitos principales.

El laboratorio debe garantizar la protección de datos de los pacientes implantando medidas para evitar que personal no autorizado pueda acceder a los datos (por ejemplo, acceso a las instalaciones, ubicación de los ordenadores, bloqueo automático, etc.). En cuanto a la entrega de informes de resultados deben considerarse todos los posibles modos de entrega (sistemas electrónicos, papel, correo electrónico o *smartphone*) para establecer las medidas de seguridad apropiadas (información disociada, encriptación, etc.).

El laboratorio debe designar las personas que están autorizadas a acceder y utilizar la información y definir niveles de seguridad basados en los perfiles de los usuarios.

Deben establecerse medidas para proteger la información, previniendo la alteración o destrucción de información por personal no autorizado. La monitorización de los sistemas electrónicos permite detectar posibles brechas de seguridad.

El laboratorio debe implementar medidas para mitigar el riesgo de degradación no intencionada o de pérdida de la información, tanto la disponible en papel como en dispositivos electrónicos.

Un requisito de la norma a considerar cuando se adquiere un sistema de gestión de la información, es que debe haber sido validado por el proveedor. Aun así, el laboratorio debe verificar y monitorizar que la transmisión de la información entre el sistema de información del laboratorio y otros sistemas tanto internos (por ejemplo, los analizadores del laboratorio) como externos (por ejemplo, el SIH) se realiza de forma correcta y que no se producen cambios no intencionados. También es preciso realizar una verificación en determinadas situaciones, como, por ejemplo, después de una actualización del *software* o después de que un equipo haya estado inactivo durante un tiempo. Esta validación/verificación debe estar documentada en un informe.

Se debe asegurar la disponibilidad y el acceso a toda la información que se genera. La ubicación del almacenaje de información debe estar claramente identificada y el acceso debe estar restringido a personal autorizado. El personal autorizado debe poder recuperar la información cuando sea necesario.

El lugar de almacenamiento de la información debe ser seguro y disponer de capacidad suficiente para la información que debe retenerse (por ejemplo, al realizar el *backup* de la información de un analizador). El laboratorio ha de definir y documentar el tiempo de retención de los documentos, cumpliendo siempre con los requisitos legales. También ha de establecer el procedimiento para identificar y destruir documentos de acuerdo al tiempo de retención definido y siempre de forma confidencial, pudiendo realizarlo el propio laboratorio o un gestor autorizado que se comprometa a ello por contrato.

## Plan de contingencia en la comunicación de resultados

La norma ISO 15189, en su punto 5.10.3, referido a la gestión del sistema de información, hace referencia a la necesidad de que el laboratorio disponga de planes de contingencia documentados para asegurar la prestación de los servicios en caso de un fallo o interrupción de los sistemas de información.

En caso de caída del SIH o del SIL, el facultativo responsable deberá activar el plan de contingencia, previamente elaborado, y hacer extensiva esta información a los responsables de los diferentes servicios hospitalarios implicados.

El plan de contingencia debe contemplar las diversas posibilidades y detallar de manera clara y concisa las actuaciones a seguir en cada caso, así como el personal responsable de las mismas [17]. Las diferentes situaciones que pueden producirse son: que no funcione el SIH o el SIL, que funcionen ambos, pero no haya comunicación entre ellos, o que no funcione ninguno de ellos. Estas situaciones pueden conllevar el uso de solicitudes en papel, su introducción manual en el SIL, así como la programación de pruebas en los diferentes equipos, impresión de copias de los resultados de los diferentes equipos junto con los pertinentes comentarios interpretativos, el envío de una copia de la solicitud y de los resultados grapados al servicio de procedencia o la impresión remota de informes en impresoras destinadas a este fin. Al finalizar la incidencia, se deben enviar los resultados desde los diferentes equipos al SIL y comprobar que, cada una de las solicitudes de las que se tiene copia, están completas.

Otras situaciones que pueden darse son, por ejemplo, el acceso indebido a la red del centro o del laboratorio (*hackers*), que no funcione la red telefónica hospitalaria, lo que implicará la necesidad de habilitar teléfonos para emergencias en el laboratorio y en los servicios con atención urgente conectados a la red eléctrica o, por ejemplo, que no funcione el tubo neumático, lo que implicará el envío de muestras a través de los celadores.

La falta de prestación de servicios también incluye averías de analizadores, equipos auxiliares, neveras, congeladores, suministro de luz, de agua o situaciones excepcionales (por ejemplo, meteorológicas) en las que el laboratorio debe garantizar la prestación del servicio a través de anexos del plan general o de diferentes planes de contingencia que afectarán sólo a determinadas áreas y en los que deben estar establecidas las prioridades, la coordinación del trabajo y la evaluación de los recursos humanos necesarios. Se detallará cómo realizar el registro de entrada manual de muestras, cómo proceder para su conservación adecuada hasta que puedan procesarse, la realización de pruebas en analizadores o equipos duplicados, o incluso puede que sea necesario, en función de la organización del laboratorio, el traslado de las muestras a otras instalaciones, asegurando que se realiza en las condiciones adecuadas.

**Figura 4:**
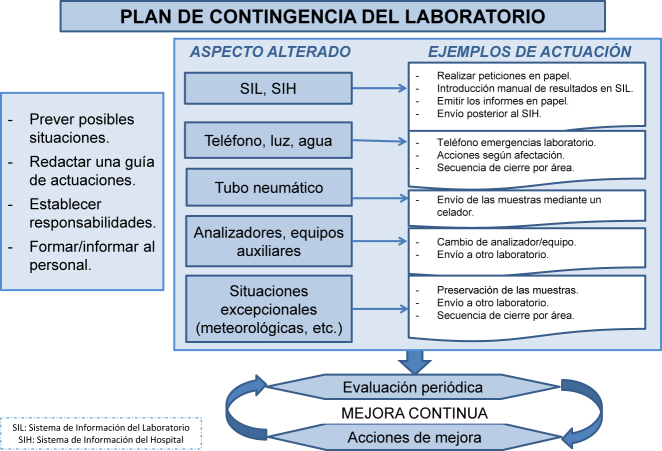
Características del plan de contingencia del laboratorio clínico.

Las características del plan de contingencia se resumen en la Figura 4.

## USO de la marca ENAC en el informe de resultados

Las entidades acreditadoras, ENAC en España, establecen las condiciones para que los laboratorios acreditados puedan hacer referencia a dicha condición, mediante el uso de la marca ENAC.

Los informes que contienen resultados de una actividad acreditada deben incluir la marca de ENAC en los informes como evidencia de su acreditación. Tal y como se describe en el documento *CEA-ENAC-01* [18], la marca ENAC resulta, actualmente, de la combinación del logotipo de ENAC (o la referencia a la condición de acreditado), la actividad acreditada y el número de acreditación.

La marca se asocia siempre al nombre o logotipo de la organización acreditada y debe aparecer al menos en la primera página del informe. Si la organización dispone de diferentes emplazamientos que realicen actividades cubiertas por la acreditación, debe identificarse el emplazamiento que ha realizado la actividad.

Cuando el informe de resultados incluya alguna actividad acreditada, puede utilizarse la marca de ENAC o bien puede hacerse referencia a la condición de acreditado con la frase “laboratorio acreditado por ENAC con acreditación n^o^ … ” tal y como se establece en el documento CEA-ENAC-01, Aptdo. 11.2. La frase debe estar escrita con el mismo tamaño y tipo de letra que el usado en el cuerpo del informe y ser legible.

Cuando no está acreditada toda la actividad incluida en el informe de resultados, para evitar confusiones y permitir reconocer fácilmente los resultados acreditados, se puede:

– Señalar con un símbolo (asterisco o similar) las magnitudes no amparadas por la acreditación e incluir una leyenda, en un lugar visible cerca de la marca, indicando que las magnitudes marcadas no están incluidas en la acreditación.
– O bien, añadir junto a cada una de las magnitudes acreditadas una marca o directamente la leyenda en la que se indique que está incluida en la acreditación. Si se ha optado por la marca, se deberá incluir también la frase explicativa en el informe.

Existen circunstancias en las que pueden emitirse informes sin marca relativos a actividades incluidas en el alcance de acreditación. En este caso, el laboratorio debe informar *a priori* a los clientes de esta circunstancia, así como de las consecuencias y recabar su aceptación. Estos casos son excepcionales, por ejemplo: cuando exista una solicitud y aceptación explícita previa del solicitante (en este caso, la organización acreditada debe informar que un informe o un certificado sin marca se considera a todos los efectos como “no acreditado”); cuando existan razones técnicas que obliguen a la organización acreditada de manera temporal a incumplir algún requisito de acreditación (en estos casos en los informes se deberá incluir una leyenda que lo indique); o cuando no sea factible el uso de la “marca” en el informe de resultados (por ejemplo, cuando el cliente visualiza los resultados a través de un portal web).

Cuando un laboratorio acreditado emite un informe que incluye actividades subcontratadas, debe quedar muy claro en el informe qué pruebas han sido subcontratadas y si están o no amparadas por una acreditación ENAC.

Ver el documento CEA-ENAC-01 para una información más completa acerca del uso de la marca ENAC.

En la Tabla 2 se resumen las principales indicaciones para el uso de la marca en los informes de resultados.

**Tabla 2:** Indicaciones principales para el uso de la marca ENAC en los informes de resultados.

– Si alguna actividad del informe está acreditada: incluir la marca o la frase, detallando el número de acreditación.
– Cuando no está acreditada toda la actividad incluida en el informe, señalar las magnitudes: – No amparadas por la acreditación, o bien, – Identificar las amparadas por la acreditación. – Siempre con frase explicativa al lado del resultado o a pie de página y detallando el número de acreditación en el informe. – Si no hay actividad acreditada en el informe, no puede aparecer la marca.
– Emisión de informes sin marca: – Por solicitud expresa del cliente. – Por incumplimiento puntual o por razones técnicas de los criterios de acreditación por parte del laboratorio. Se incluirá frase explicativa en el informe. – Cuando no sea factible su uso (visualización por portal web).
– Actividad subcontratada: – Actividad acreditada subcontratada a un laboratorio acreditado: indicar claramente qué pruebas han sido subcontratadas. – Actividad no acreditada subcontratada a un laboratorio acreditado: puede incluirse una frase indicando el número de acreditación del laboratorio subcontratado, pero no se puede utilizar la marca.
– Alcance flexible: las pruebas acreditadas se incluyen, por categorías, en la Lista de Análisis Acreditados.
